# A temporal model of neural activity and VSD response in V1

**DOI:** 10.1186/1471-2202-13-S1-P180

**Published:** 2012-07-16

**Authors:** Jean-Luc R Stevens, James A Bednar

**Affiliations:** 1 Institute for Adaptive and Neural Computation, University of Edinburgh, EH8 9AB, UK

## 

Mechanistic developmental models of the primary visual cortex (V1) in mammals have been able to replicate many of the large-scale spatial features of V1 neurons from experiments, such as their spatial receptive fields and the spatial organization into maps in V1 (reviewed in ref [1]). However, the models have previously been formulated at a very abstract level that does not account for the detailed, transient time course of neural responses. Conversely, there are a number of detailed, large-scale spiking models of the adult visual cortex, but these have not explained the development of feature preferences and feature maps, relying instead on prespecified patterns of connectivity. Here we present a new temporally and spatially calibrated model of cortical activity using rate-based units that could help unify these different types of explanation and levels of modelling. The model is called TCAL (Temporally CALibrated), and is a small variant on the GCAL model from the LISSOM family [1].

**Figure 1:**
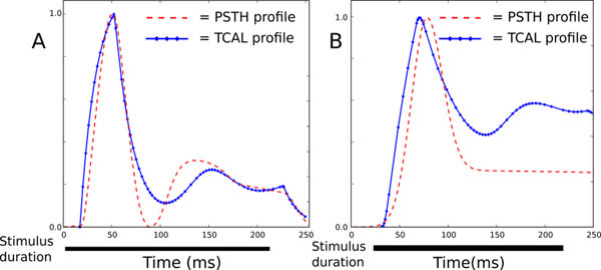
Step temporal response of TCAL model LGN cells (A, plotted against macaque LGN PSTH data from ref [2]) and model V1 cells (B, plotted against macaque V1 PSTH data from ref [3]).

Compared to GCAL, the only change to the model mechanisms is to add hysteresis to the model LGN and V1 units. Hysteresis allows the damping of temporal responses to be controlled with one time-constant parameter per sheet. These two new parameters were set first for the LGN and then for V1to match results from electrophysiological recordings. Both onset and offset responses are matched against experimentally recorded peristimulus time histograms (PSTHs) for LGN [2] and cortical [3] neurons using the Invariant Response Description model. Despite the two orders of magnitude difference in time scales between GCAL and TCAL and the minimal change to the GCAL rate-based mechanisms, the time course of responses is already a remarkably close match (see Figure 1).

The transient responses of LGN neurons in TCAL are due to lateral inhibition between LGN/RGC cells already present in GCAL, but originally for purposes of contrast gain control. The transient responses at the V1 level are partly inherited from LGN but also reflect lateral connectivity in V1, originally for the purposes of map development. TCAL thus shows how the observed transient response properties can arise from the same mechanisms that lead to map development in mechanistic Hebbian models [1]. After calibrating the afferent response delays and distance-dependent lateral connection delays, TCAL can now be used to predict the spatial and temporal time course of voltage-sensitive-dye (VSD) responses to spatiotemporal visual stimuli, and allows the detailed dynamics of perceptual phenomena to be studied even without spiking models.
